# Association between posterior occlusal pairs loss and severe sarcopenia in community-dwelling Chinese older adults: A cross-sectional study

**DOI:** 10.1097/MD.0000000000043371

**Published:** 2025-07-11

**Authors:** Wei Feng, Kangkang Chen, Qifeng Chen, Laichao Xu

**Affiliations:** a Department of Orthodontics, Shaoxing Stomatological Hospital, Shaoxing, Zhejiang Province, China; b Department of Non-communicable Diseases Control and Prevention, Shaoxing Center for Disease Control and Prevention, Shaoxing, Zhejiang Province, China; c Administrative Office, Shaoxing Center for Disease Control and Prevention, Shaoxing, Zhejiang Province, China.

**Keywords:** community, nutrition, older adults, severe sarcopenia, tooth loss

## Abstract

Individuals with severe sarcopenia are at a higher risk of adverse health events than those with sarcopenia alone. However, due to the limited number of studies, the condition remains poorly understood. To date, few studies have examined the association between tooth loss and severe sarcopenia. Therefore, we conducted a cross-sectional study to address this gap. Community residents who were 60 years and older, and had attended a local government basic health checkup from March to October in 2022 were recruited. Severe sarcopenia was defined according to the Asian Working Group for Sarcopenia 2019. Loss of occlusal pairs (14 total pairs: 6 anterior occlusal pairs, 8 posterior occlusal pairs [POPs]) was quantified through dental examinations. Logistic regressions were used to evaluate the association between loss of occlusal pairs and severe sarcopenia, adjusting for potential confounding factors. A total of 1421 older adults (42.6% men, 71.4 ± 6.8 years old) were enrolled in this study. The prevalence of severe sarcopenia was 8.3%. Multivariate analyses showed that each additional POPs loss increased severe sarcopenia risk (adjusted OR = 1.17, 95% CI = 1.06–1.28, *P* = .001), while anterior occlusal pairs loss showed no association (*P* = .982). From the angle of population group, significant increase in severe sarcopenia was only found in older adults with the highest levels of missing POPs compared to those without (Δ high vs low loss: 12.45% (*P* < .001); Δ high vs low–medium loss: 10.14% (*P* < .001); Δ high vs medium–high loss: 9.85% (*P* < .001)). The identification and management of POPs loss, high loss in particular, could be important in preventing severe sarcopenia in community-dwelling older adults.

## 1. Introduction

Severe sarcopenia was formally introduced in the Asian Working Group for Sarcopenia 2019.^[1]^ It is characterized by a loss of muscle mass, muscle strength and physical performance with age. Most of the available evidence indicates that severe sarcopenia can significantly reduce individuals’ physical independence.^[2,3]^ Currently, the global prevalence of severe sarcopenia ranges from 2% to 9%,^[4]^ and as populations are aging worldwide, the number of people living with this disorder is expected to increase, which means more people will be at higher risks of low quality of life, falls and bone fracture. Beyond age-related factors, patients with certain clinical conditions such as acute heart failure are more prone to develop severe sarcopenia,^[5]^ which in turn is independently associated with a poor prognosis.^[5–7]^ Thus, identifying factors that are beneficial for the prevention and treatment of severe sarcopenia is important, wherein oral health shows a great potential.

Poor oral health was shown to be associated with multiple diseases such as depression,^[8]^ chronic illness,^[9]^ and cancer.^[10]^ Poor oral health can affect masticatory function and food intake,^[11]^ resulting in malnutrition which is one of the causes of severe sarcopenia.^[12]^ There are several indicators used for assessing poor oral health, including dental caries, periodontal diseases, tooth loss, etc.^[13]^ Tooth loss is typically the endpoint of oral disease, and unlike other indicators, counting the number of present teeth is a simpler and more objective measurement, leading to research conclusions being realistic and reliable.

So far, no previous studies have been conducted to investigate the relationship between tooth loss and severe sarcopenia. Given the limited number of studies on severe sarcopenia, and the resulting narrow view of this classification relative to sarcopenia, improving the recognition of this disease is necessary. Thus, we conducted this study to explore whether severe sarcopenia is associated with tooth loss, especially loss of occlusal pairs, in order to explore potential associations that may inform prevention strategies.

## 2. Materials and methods

### 2.1. Study participants

This is a cross-sectional study conducted by Shaoxing Center for Disease Control and Prevention from November 2022 to December 2022. Community-based sampling was carried out to recruit participants in 2 subdistricts (Maan and Donghu) in Shaoxing city. In 2022, Maan had a population of 39,351, of which nearly 26% were older adults (≥60 years old), and Donghu had a population of 42,768 with a population aging rate of 29%. Community residents who were 60 years and older, and had attended a local government basic health checkup from March to October in 2022 were allowed to participate in this study. Additionally, 2 exclusion criteria were set: participants who had implanted electronic devices and metal implants; and participants were unable to perform the requested tests due to the physical and/or mental impairment. The candidates were informed of the date and venue of questionnaire survey and physical tests, by phone, in advance. Participants’ basic information was exported from basic health checkup records, and other information we needed was collected through face-to-face interviews by trained researchers.

### 2.2. Ethics approval and consent to participate

This study was conducted with the approval of the Research Ethics Committee of Shaoxing Center for Disease Control and Prevention, and all subjects provided written informed consent. All methods were performed in accordance with the Declaration of Helsinki.

### 2.3. Severe sarcopenia

As participants included in this study were from China, Asian Working Group for Sarcopenia 2019 algorithm was used to diagnose severe sarcopenia, which is characterized by low muscle strength, low physical performance, and low muscle mass.^[1]^ To evaluate muscle strength, a digital handgrip dynamometer (EH101; Xiangshan Inc., Guangdong, China) was used. The participants were asked to stand upright with elbows fully extended, and arms parallel to their bodies. After the researchers giving the instruction, each participant started to squeeze the handle with maximum effort using dominant hand. The measurements were taken 3 times, and the highest value was taken as the final result. Grip strength of <28 kg for men and <18 kg for women was defined as low muscle strength. Usual gait speed was measured to assess physical performance. Participants were instructed to walk over a distance of 6 m at a normal pace, without acceleration or deceleration. A 6-m gait speed is equal to 6 m divided by the recorded time. The participants performed 2 trials, and the average value was calculated as the recorded speed. A gait speed of <1.0 m/s was defined as low physical performance. We used a bioelectrical impedance analysis device (MC-780MA; TANITA Inc, Japan) to measure the appendicular skeletal muscle mass; its value was normalized by the square of the height (in meters). The suggested bioelectrical impedance analysis cutoff values for low muscle mass were <7.0 kg/m^2^ in men and <5.7 kg/m^2^ in women.

### 2.4. Occlusal pairs loss

The total number of occlusal pairs in healthy adults was defined as 14, including 6 anterior occlusal pairs (AOPs) and 8 posterior occlusal pairs (POPs). The trained researchers counted the number of present teeth, and recorded the location of each missing tooth. Furthermore, the severity of occlusal pairs loss was grouped into 4 classifications (i.e., high, medium–high, low–medium and low loss) according to interquartile range.

### 2.5. Covariates

Because the number of participants with severe sarcopenia was limited, we carefully selected covariates which were shown to be clinically relevant based on a review of literature. The covariates included sociodemographic factors (age and gender); consumption of dairy, which was grouped into (per day, per week, per month, and never); smoking status and alcohol consumption, which were categorized into 3 groups (never, current, and previous); sleeping duration, sedentary time, mean number of comorbidities, and hemoglobin A1c, which were used as continuous variables. Variables such as physical activity level and use of prosthetic teeth were not included in the analysis due to data unavailability, although they may be important confounders.

### 2.6. Statistical analysis

Descriptive statistics were performed stratified by severe sarcopenia status. Continuous variables with a normal distribution were calculated as means ± standard deviation; continuous variables with a non-normal distribution were calculated as median (interquartile range); categorical variables were presented as numbers (percentages). Differences between participants with and without severe sarcopenia were evaluated using the independent *t*-test, Wilcoxon rank sum test, chi-square test, and Fisher exact test as appropriate. Logistic regression analyses were used to identify independent factors related to severe sarcopenia, via a stepwise selection procedure. Three models were constructed: Model 1 was adjusted for loss of occlusal pairs, age, and gender; Model 2 was additionally adjusted for consumption of dairy; and Model 3 was additionally adjusted for alcohol consumption, smoking groups, sleeping duration, sedentary time, mean number of comorbidities, and hemoglobin A1c. odds ratios and 95% confidence intervals were reported. Also, to identify population subgroups that warrant targeted public health interventions, we examined the prevalence of severe sarcopenia across populations with varying degrees of occlusal pairs loss using chi-square tests, with the significance level adjusted for multiple comparisons via the Bonferroni correction (α = 0.05/6 = 0.0083). All analyses were performed with SPSS v20.0 (SPSS Statistics; IBM, Armonk). Statistical significance was set at 2-sided *P *< .05.

## 3. Results

### 3.1. Characteristics of participants

A total of 1421 older adults (42.6% men, 71.4 ± 6.8 years old) were included in this study, among whom 8.3% were diagnosed with severe sarcopenia. The mean ages of participants with and without severe sarcopenia were 79.5 and 70.7 years, respectively. Participants with severe sarcopenia had a higher percentage of current smokers and a greater mean number of comorbidities. In addition, compared to those without severe sarcopenia, these participants exhibited a higher number of missing occlusal pairs in total, including both POPs and AOPs. Notably, the proportion of complete occlusal pairs loss was 55.9% among those with severe sarcopenia, compared to only 18.6% in those without (Table 1).

**Table 1:** Baseline characteristics in 1421 participants.

Variable	Overall (n = 1421)	Non-severe sarcopenia (n = 1303)	Severe sarcopenia (n = 118)	*P*-value
Age, yr	71.4 ± 6.8	70.7 ± 6.3	79.5 ± 6.9	**<.001**
Males, n (%)	606 (42.6)	560 (43.0)	46 (39.0)	.401
Height, cm	158.4 ± 8.4	158.7 ± 8.4	155.0 ± 7.8	**<.001**
Weight, kg	60.2 ± 10.2	61.0 ± 10.0	51.2 ± 7.0	**<.001**
Smoking groups, n (%)				
Never	1001 (70.4)	916 (70.3)	85 (72.0)	**.034**
Current	298 (21.0)	268 (20.6)	30 (25.4)	
Previous	122 (8.6)	119 (9.1)	3 (2.5)	
Alcohol consumption, n (%)				
Never	855 (60.2)	775 (59.5)	80 (67.8)	.085
Current	492 (34.6)	456 (35.0)	36 (30.5)	
Previous	74 (5.2)	72 (5.5)	2 (1.7)	
Sleeping duration, h	6.8 ± 2.0	6.8 ± 1.9	6.7 ± 2.3	.742
Sedentary time, h	3.0 ± 2.4	3.0 ± 2.4	3.2 ± 2.6	.472
Consumption of dairy, n (%)				
Per day	230 (16.2)	218 (16.7)	12 (10.2)	.205
Per week	183 (12.9)	170 (13.0)	13 (11.0)	
Per month	188 (13.2)	169 (13.0)	19 (16.1)	
Never	820 (57.7)	746 (57.3)	74 (62.7)	
Mean number of comorbidities	1.8 ± 0.8	1.8 ± 0.8	1.9 ± 0.8	**.038**
Haemoglobin A1c (%)	5.7 ± 0.9	5.7 ± 0.9	5.6 ± 0.8	.916
Loss of occlusal pairs	7.4 ± 5.1	7.1 ± 5.0	10.9 ± 4.5	**<.001**
Loss of anterior occlusal pairs	2.7 ± 2.5	2.6 ± 2.4	4.3 ± 2.3	**<.001**
Loss of posterior occlusal pairs	4.7 ± 3.0	4.5 ± 3.0	6.6 ± 2.4	**<.001**
Complete occlusal pairs loss, n (%)	308 (21.7)	242 (18.6)	66 (55.9)	**<.001**
Skeletal muscle mass				
ASM/height^2^	6.9 ± 1.1	7.0 ± 1.1	5.8 ± 0.6	**<.001**
ASM/height^2^ (<7.0 in men and <5.4 in women), %	198 (13.9)	80 (6.1)	118 (100.0)	**<.001**
Muscle strength				
Grip strength, kg	22.5 ± 9.4	23.2 ± 9.3	14.6 ± 6.0	**<.001**
Grip strength (<28 kg in men and < 18 kg in women), %	709 (49.9)	591 (45.4)	118 (100.0)	**<.001**
Physical performance				
6-m usual gait speed, m/s	1.0 ± 0.3	1.1 ± 0.3	0.8 ± 0.2	**<.001**
Gait speed (<1.0 m/s in men and women), %	593 (41.7)	475 (36.5)	118 (100.0)	**<.001**

ASM = appendicular skeletal muscle mass.

Bold values indicate statistically significant results (*P* < .05).

### 3.2. Association of missing occlusal pairs with severe sarcopenia

The association between severe sarcopenia and the number of missing total occlusal pairs is presented in Table 2. In the first model, the OR for severe sarcopenia increased to 1.08 (95% CI: 1.02–1.13, *P* = .003) for each additional missing occlusal pair. After adjusting for consumption of dairy in model 2, the OR remained unchanged. With further adjustments for alcohol consumption, smoking, sleeping duration, sedentary time, comorbidity, and hemoglobin A1c in model 3, the OR increased slightly to 1.09 (95% CI: 1.03–1.14, *P* = .002).

**Table 2:** Multivariate logistic regression models showing the associations between loss of occlusal pairs and severe sarcopenia (N = 1421).

	Model 1		Model 2		Model 3	
	OR	*P*-value	OR	*P*-value	OR	*P*-value
Age	1.19 (1.15–1.23)	**<.001**	1.20 (1.15–1.24)	**<.001**	1.19 (1.15–1.23)	**<.001**
Gender						
Male	(Reference)					
Female	1.94 (1.25–3.02)	**.003**	1.89 (1.21–2.93)	**.005**	1.81 (1.10–2.99)	**.021**
Loss of occlusal pairs	1.08 (1.02–1.13)	**.004**	1.08 (1.02–1.13)	**.005**	1.09 (1.03–1.14)	**.002**
Consumption of dairy			1.28 (1.05–1.56)	**.016**	1.32 (1.08–1.61)	**.007**
Alcohol consumption						
Never	(Reference)					
Current					0.83 (0.51–1.34)	.440
Previous					0.36 (0.08–1.75)	.201
Smoking groups						
Never	(Reference)					
Current					1.47 (0.85–2.55)	.166
Previous					0.32 (0.09–1.15)	.081
Sleeping duration					0.96 (0.87–1.06)	.434
Sedentary time					0.98 (0.90–1.06)	.547
Mean number of comorbidities					1.35 (1.05–1.75)	**.021**
Haemoglobin A1c					1.06 (0.84–1.34)	.642

Model 1: adjusted for loss of occlusal pairs, age, and gender, Model 2: additionally adjusted for consumption of dairy, Model 3: additionally, adjusted for alcohol consumption, smoking groups, sleeping duration, sedentary time, mean number of comorbidities, and hemoglobin A1c. Bold values indicate statistically significant results (*P* < .05).

When occlusal pairs were categorized into the AOPs and POPs, the number of missing POPs remained significantly associated with severe sarcopenia in the multivariable logistic regression analysis (OR = 1.17, 95% CI: 1.06–1.28, *P* = .001) (Table 3). In contrast, no significant association was observed between the number of missing AOPs and severe sarcopenia (OR = 1.00, 95% CI = 0.88–1.14, *P* = .982).

**Table 3:** Multivariate logistic regression models showing the associations between loss of posterior occlusal pairs and severe sarcopenia (N = 1421).

Variables	OR	*P*-value
Age	1.20 (1.15–1.24)	**<.001**
Gender		
Male		
Female	1.79 (1.09–2.96)	**.023**
Consumption of dairy	1.31 (1.07–1.60)	**.008**
Alcohol consumption		
Never		
Current	0.82 (0.51–1.34)	.433
Previous	0.36 (0.08–1.75)	.208
Smoking groups		
Never		
Current	1.47 (0.85–2.54)	.167
Previous	0.32 (0.09–1.15)	.081
Sleeping duration	0.96 (0.87–1.06)	.454
Sedentary time	0.97 (0.90–1.06)	.518
Mean number of comorbidities	1.34 (1.04–1.74)	**.024**
Haemoglobin A1c	1.05 (0.83–1.33)	.689
Number of missing occlusal pairs		
AOPs	1.00 (0.88–1.14)	.982
POPs	1.17 (1.06–1.28)	**.001**

Model was adjusted for age, gender, consumption of dairy, alcohol consumption, smoking, sleeping duration, sedentary time, mean number of comorbidities, hemoglobin A1c, AOPs, and POPs. Bold values indicate statistically significant results (*P* < .05).

AOPs = anterior occlusal pairs, POPs = posterior occlusal pairs.

Subgroup analyses were performed based on age and gender. After adjustment for other confounding factors, participants aged 60 to 69, 70 to 79, and being male were more likely to suffer from severe sarcopenia, with an OR of 1.49 (95% CI = 1.11–1.20), 1.14 (95% CI = 1.01–1.28), and 1.29 (95% CI = 1.09–1.53), respectively (Table 4).

**Table 4:** Stratification analyses for the relationship between severe sarcopenia and loss of POPs.

Variables	OR	*P*-value
Age (yr)		**<.001**
60–69	1.49 (1.11–1.20)	**.008**
70–79	1.14 (1.01–1.28)	**.033**
≥80	1.18 (0.99–1.40)	.057
Gender		
Male	1.29 (1.09–1.53)	**.003**
Female	1.09 (0.98–1.22)	.129

Model was adjusted for consumption of dairy, mean number of comorbidities, and age (for gender stratification analysis), or gender (for age stratification analysis). Bold values indicate statistically significant results (*P* < .05).

POPs = posterior occlusal pairs.

### 3.3. Prevalence of severe sarcopenia by POPs loss severity

**Figure 1.**
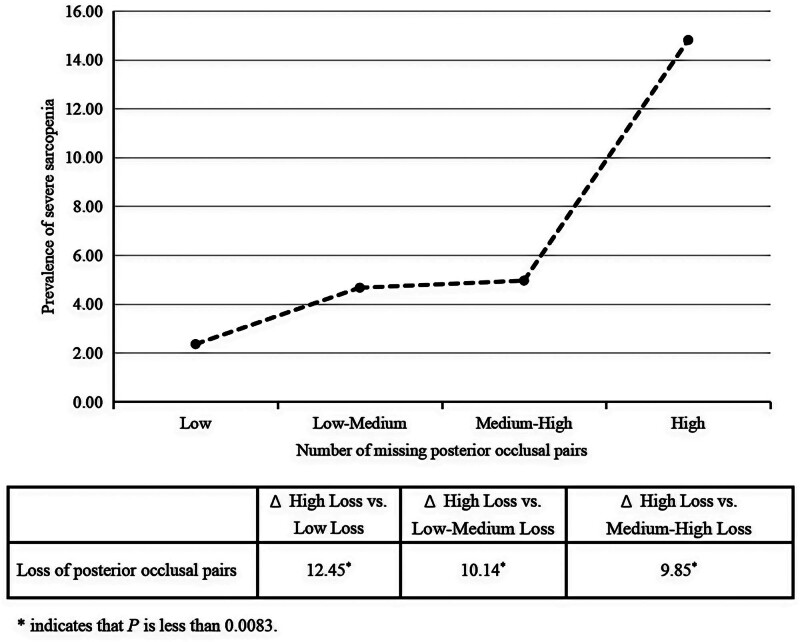
Prevalence of severe sarcopenia by the severity of the missing number of POPs. Horizontal axis shows older adults grouped into 4 classifications according to their missing POPs levels, that is: high, medium–high, low–medium and low loss. These correspond, respectively, to the 4th (75th–100th percentile), 3rd, 2^nd^, and 1st (0th–25th percentile) quartile of POPs loss. Vertical axis shows the prevalence of severe sarcopenia. Older adults with different degrees of POPs loss were compared with each other. A difference exceeding at least 5 points, can be considered as a clinically meaningful difference and was listed in the above table. POPs = posterior occlusal pairs.

As missing POPs was found to be the risk factor associated with severe sarcopenia in multivariate analysis, we reported prevalence of severe sarcopenia by POPs loss severity, in order to identify population subgroups with the highest burden of severe sarcopenia, thereby prioritizing targets for public health interventions. In terms of the changes in prevalence of severe sarcopenia, only difference between older adults with the highest levels of missing POPs and those without was clinically meaningful (Δ high vs low loss: 12.45% [*P* < .001]; Δ high vs low–medium loss: 10.14% [*P* < .001]; Δ high vs medium–high loss: 9.85% [*P* < .001]) (Fig. 1).

## 4. Discussion

To our knowledge, this was the first study to investigate the influence of occlusal pairs loss on severe sarcopenia in community-dwelling older adults. We observed that older adults with severe sarcopenia, particularly men and those aged 60 to 79, tended to have fewer POPs, whereas AOPs loss was not associated with severe sarcopenia. In terms of prevalence of severe sarcopenia, significant increase was only found in older adults with the highest levels of missing POPs compared to those without.

A meta-analysis by Petermann-Rocha et al^[4]^ concluded that the global prevalence of severe sarcopenia was 2% to 9%. Additionally, among Asian community-dwelling older adults, the prevalence of severe sarcopenia was reported to range from 1.2% to 13.7% based on the AWGS2019 criteria.^[2,14–17]^ In the present study, the prevalence of severe sarcopenia in the Chinese counterparts was 8.3%, within the range reported in previous academic literature. With regard to edentulism, nearly 30% of older adults aged 65 to 74 have lost all of their teeth worldwide.^[18]^ Another study conducted in 16,501 adults in China reported that the prevalence of edentulism was 25.6% for 65 to 74 age group.^[19]^ In the present study, the proportion of complete occlusal pairs loss in participants with the mean age of 71.4 was 21.7%, which is slightly lower than previous studies. It may be from the fact that as a second-tier city in China, Shaoxing has the better oral public health services. In general, the good consistency in the prevalence of severe sarcopenia and edentulism suggests the reliability and generalization of our findings.

Consistent with our findings, several studies conducted in China have shown that poor dental status is linked to sarcopenia among older adults. Lin et al^[20]^ examined 2850 community-dwelling older adults in Nanjing and found that individuals with fewer than 5 POPs had a significantly greater prevalence of sarcopenia compared to those with 5 or more POPs. Another cohort study of older adults in rural eastern China (n = 529) also showed severe tooth loss predicted higher sarcopenia incidence over 4 years (OR = 1.80; 95% CI = 1.02–3.21).^[21]^ Additionally, a large Chinese analysis involving 4149 adults found a significant association between the number of teeth (β = −0.327, 95% CI: −0.471 to −0.237) and sarcopenia.^[22]^ Our study further extended these findings, demonstrating that this relationship also applies to severe sarcopenia. However, not all research supports this association. A cross-sectional study of 1494 older adults in suburban Shanghai and Tianjin found no significant correlation between self-reported tooth loss and sarcopenia or muscle mass.^[23]^ This discrepancy may be attributed to differences in oral health assessment methods (self-reported tooth loss vs clinical dental examination) and variations in sample characteristics such as socioeconomic factors, dietary habits, or access to healthcare services. Overall, the majority of evidence, including the Chinese studies referenced above and our results, supports a positive relationship between poor dental health – particularly the loss of POPs – and an increased risk of sarcopenia among older adults.

A possible explanation for the observed association between loss of POPs and severe sarcopenia involves their essential role in mastication, as POPs are crucial for effective chewing and grinding of food.^[24]^ A reduced number of POPs typically results in decreased chewing efficiency among older adults, leading to insufficient nutritional intake – a well-established risk factor for sarcopenia.^[12]^ Additionally, tooth loss may alter dietary patterns, resulting in nutritional imbalance. For example, Toniazzo et al^[25]^ reported that tooth loss was associated with reduced enjoyment and consumption of fruits, vegetables, and fiber-rich foods. These food groups are the primary dietary sources of short-chain fatty acids, which significantly influence muscle biology.^[26,27]^ Indeed, Xia et al^[22]^ highlighted that nutritional status partially mediated the relationship between tooth count and sarcopenia, supporting the notion that impaired mastication resulting from tooth loss could lead to malnutrition and subsequent muscle loss.

Systemic chronic inflammation is considered an important contributor to the development of sarcopenia.^[26,28]^ Considering that tooth loss in older adults is mainly attributed to periodontitis,^[29]^ one might expect that periodontal inflammatory levels mediate the relationship between tooth loss and severe sarcopenia. In addition, recent evidence suggests that age-related musculoskeletal decline may also involve cellular senescence and the senescence-associated secretory phenotype, which drives chronic inflammation and tissue degeneration. As highlighted by Li et al,^[30]^ the accumulation of senescent cells and senescence-associated secretory phenotype in skeletal tissues can disrupt tissue homeostasis and accelerate bone aging. While their findings focus primarily on bone, similar senescence-associated mechanisms may also contribute to muscle loss, potentially linking oral health deterioration to systemic aging processes. A particular strength of our study is that we counted the location of each missing tooth. According to our findings, POPs loss rather than AOPs loss was associated with severe sarcopenia, suggesting no or only a little contribution of inflammation induced by tooth loss to developing severe sarcopenia. In contrast, 1 possible reason is that the function of the AOPs is far less powerful than that of the POPs in terms of crushing and grinding food.

Another key finding which has never been reported is that the significant increase in prevalence of severe sarcopenia was only observed in the group with the highest levels of missing POPs. In terms of nutritional status, this finding could be attributed to 2 aspects. On the one hand, having edentulism or severe POPs loss may make it harder for older adults to eat, subsequently reducing the quality and quantity of nutritional intake.^[31]^ On the other hand, both tooth loss and sarcopenia are chronic diseases, which are worsened by time. Therefore, it is possible that severe sarcopenia caused by tooth loss are most likely to occur at the time of complete tooth loss. Indeed, many previous studies have reported that there was a longitudinal association between poor oral health, walking speed decline, sarcopenia, and physical frailty.^[19,32–34]^ Therefore, our findings particularly highlight the importance of the management of severe POPs loss in community-dwelling older adults, in order to prevent severe sarcopenia.

The present study has several limitations: all older adults were asked to walk and stand independently in the process of sarcopenia diagnosis, so those with poor physical performance such as bedridden patients were less likely to participate in this research. Also, participation was voluntary, and therefore health-conscious individuals with good activity levels may be dominant in this research. Therefore, selection bias may exist in our study. When assessing the condition of participants’ teeth, the use of dentures was not recorded. However, national data suggest that approximately 63% of older adults with missing teeth in China wear dentures,^[35]^ indicating that a substantial proportion rely on prosthetic replacements. As dentures can partially restore occlusal function, their use may mitigate the impact of occlusal pairs loss on nutrient intake by improving chewing efficiency.^[36]^ Similarly, physical activity, a known factor affecting muscle mass and strength, was not assessed in our analysis.^[1]^ The absence of these variables, along with the limited number of severe sarcopenia cases restricting the inclusion of covariates in multivariate models, may have introduced residual confounding. Future studies should consider including these and other relevant factors to better clarify the observed associations. As with all cross-sectional studies, only associations – not causal relationships – can be established. Therefore, it remains unclear whether tooth loss contributes to severe sarcopenia or vice versa.

In conclusion, our study suggests that tooth loss – particularly the loss of POPs – is significantly associated with severe sarcopenia in community-dwelling older adults. These findings underscore the potential role of dental health, especially in cases of extensive POPs loss, in preventing severe sarcopenia. However, given the cross-sectional design, future longitudinal clinical studies are needed to determine causality and clarify the direction of this association.

## Acknowledgments

We are grateful to staffs from Yuecheng Center for Disease Control and Prevention and Keqiao Center for Disease Control and Prevention for helping us collect the data.

## Author contributions

**Conceptualization:** Laichao Xu.

**Data curation:** Kangkang Chen, Qifeng Chen.

**Formal analysis:** Wei Feng, Kangkang Chen.

**Writing – original draft:** Wei Feng.
